# Identification of an optimal prolactin threshold to determine prolactinoma size using receiver operating characteristic analysis

**DOI:** 10.1038/s41598-021-89256-7

**Published:** 2021-05-07

**Authors:** Bianca M. Leca, Maria Mytilinaiou, Marina Tsoli, Andreea Epure, Simon J. B. Aylwin, Gregory Kaltsas, Harpal S. Randeva, Georgios K. Dimitriadis

**Affiliations:** 1 Warwickshire Institute for the Study of Diabetes, Endocrinology and Metabolism (WISDEM), University Hospitals Coventry and Warwickshire NHS Trust, Coventry, CV2 2DX UK; 2 1st Department of Propaedeutic Internal Medicine, Endocrine Unit, National and Kapodistrian, University of Athens, 11527 Athens, Greece; 3 Achilles Information Limited, Abingdon, OX14 4SH UK; 4 Department of Endocrinology ASO/EASO COM, King’s College Hospital NHS Foundation Trust, Denmark Hill, London, SE5 9RS UK; 5 Division of Reproductive Health, Warwick Medical School, University of Warwick, Coventry, CV4 7AL UK; 6 Aston Medical Research Institute, Aston Medical School, Aston University, Birmingham, B4 7ET UK; 7 Department of Obesity, Immunometabolism and Diabetes, School of Life Course Sciences, King’s College London, London, SE1 9RT UK

**Keywords:** Endocrinology, Endocrine system and metabolic diseases

## Abstract

Prolactinomas represent the most common type of secretory pituitary neoplasms, with a therapeutic management that varies considerably based on tumour size and degree of hyperprolactinemia. The aim of the current study was to evaluate the relationship between serum prolactin (PRL) concentrations and prolactinoma size, and to determine a cut-off PRL value that could differentiate micro- from macro-prolactinomas. A retrospective cohort study of 114 patients diagnosed with prolactinomas between 2007 and 2017 was conducted. All patients underwent gadolinium enhanced pituitary MRI and receiver operating characteristic (ROC) analyses were performed. 51.8% of patients in this study were men, with a mean age at the time of diagnosis of 42.32 ± 15.04 years. 48.2% of the total cohort were found to have microadenomas. Baseline serum PRL concentrations were strongly correlated to tumour dimension (r = 0.750, *p* = 0.001). When performing the ROC curve analysis, the area under the curve was 0.976, indicating an excellent accuracy of the diagnostic method. For a value of 204 μg/L (4338 mU/L), sensitivity and specificity were calculated at 0.932 and 0.891, respectively. When a cut off value of 204 μg/L (4338 mU/L) was used, specificity was 93.2%, and sensitivity 89.1%, acceptable to reliably differentiate between micro- and macro- adenomas.

## Introduction

Hyperprolactinemia represents an increase in circulating prolactin (PRL) levels, producing hypogonadism and infertility in both sexes^1^. With a broad aetiology, hyperprolactinemia may arise through different mechanisms, and can have physiological, pathological, or pharmacological causes^2^. After ruling out pregnancy, hypothyroidism, uraemia, hepatic insufficiency and use of medications that could increase serum PRL levels, prolactinomas are the most common cause of chronic hyperprolactinemia^3–5^. Apparently high levels of prolactin may be due to excess circulating prolactin complexes (‘macroprolactin’) which need to be differentiated from true hyperprolactinemia. Increased prolactin may also rise due to acute medical stress and therefore diagnostic levels of prolactin should be ideally taken after intravenous cannulation^6^.

Prolactinomas represent the most common secretory pituitary neoplasms, accounting for 57% of all pituitary adenomas^2–5,7^. The prevalence is higher in women, with a peak in those aged 16 to 48 years. After the fifth decade of life frequency becomes equal in both genders^1–3,8^.

Based on their size, prolactinomas are classified as either microadenomas measuring < 1 cm, and macroadenomas ≥ 1 cm^7^. Microadenomas represent 80% of prolactinomas, while malignant neoplasms are uncommon. In addition, approximately 80% of prolactinomas in men are macroadenomas, while women present more frequently with microadenomas^8^.

Generally, the degree of hyperprolactinemia correlates with the size of the prolactinoma and increase in tumour size enhancing the synthesis and release of PRL^2^. For microadenomas, PRL usually ranges from 94 to 188 μg/L (2000 to 4000 mU/L). However, microprolactinomas may present with prolactin concentration < 94 μg/L (2000 mU/L) or reach occasionally up to 470 μg/L (10,000 mU/L)^2,3,5^. Macroprolactinomas commonly present with PRL concentrations > 235 μg/L (5000 mU/L)^1,9^. Furthermore, the “hook effect” should be considered in patients with normal or mildly elevated prolactin concentration and large pituitary adenomas (≥ 3 cm), particularly in laboratories using older generation PRL assays^3^. When hyperprolactinemia is caused by hypothalamic damage or pituitary stalk compression (pseudoprolactinoma), PRL concentration is usually < 94 μg/L (2000 mU/L)^1,3^. Interpretation of PRL results should always be made according to assay specific reference ranges.

After excluding other causes of elevated prolactin, imaging of the sellar region should be performed, with gadolinium enhanced pituitary magnetic resonance imaging (MRI) representing the gold standard radiological method for diagnosing prolactinomas^2,5^.

The main goal of treatment for hyperprolactinemia is to restore and maintain normal gonadal function, and its management depends on the underlying cause^2,5^. The available therapeutic options for prolactinomas consist in pharmacotherapy with dopamine agonists (DAs), surgery and radiation therapy^5^. Current guidelines recommend cabergoline as a first line treatment, with usual doses of 0.5 to 3 mg weekly^2,5^.

Existing guidelines suggest that with careful clinical and biochemical follow-up, DA therapy may be tapered or discontinued after at least 2 years of treatment, if PRL concentration remains normal and there is no recurrent tumour on MRI^5^.

The aim of the current study was to evaluate the relationship between serum PRL concentrations and prolactinoma size, and to determine a cut-off PRL value that could reliably differentiate between micro- and macroadenomas before radiological assessment.

## Materials and methods

A retrospective cohort study of 114 patients diagnosed with prolactinomas at a single academic institution between 2007 and 2017 was conducted.

The diagnosis of prolactinoma was made based on all of the following: (1) radiological evidence of pituitary adenoma, (2) sustained hyperprolactinemia (PRL concentration greater than the upper normal limit in two different samples collected in two different days), and (3) clinical symptoms of hyperprolactinemia. Patients who presented with other causes of elevated serum prolactin (pregnancy, lactation, hypothyroidism, polycystic ovary syndrome, uraemia, hepatic insufficiency, use of medications that could increase PRL levels) were excluded from the analysis.

### Blood samples

Pre-treatment serum concentration of PRL was determined using Prolactin I immunoassay on Roche Elecsys in patients diagnosed before 2010, and Prolactin II immunoassay on Cobase in those diagnosed after. Both tests use two monoclonal antibodies specifically directed against human prolactin, the key difference being the reduced cross-reactivity with macroprolactin complexes for the second-generation assay. When switching between assays, no change in bias was observed, and the reference range remained unchanged. Serum prolactin reference range for our hospital is < 24 μg/L (501 mU/L) in women, and < 19 μg/L (401 mU/L) in men.

In addition, FSH, LH, and oestradiol levels in pre-menopausal women and FSH, LH, and testosterone levels in men were evaluated. Liver, kidney, and thyroid function tests were performed, and pregnancy was excluded in all females of childbearing potential.

All blood samples were collected in the morning, from an antecubital vein, with participants fasting for at least 8 h, in a resting state.

### MRI scanning technique

**Figure 1:**
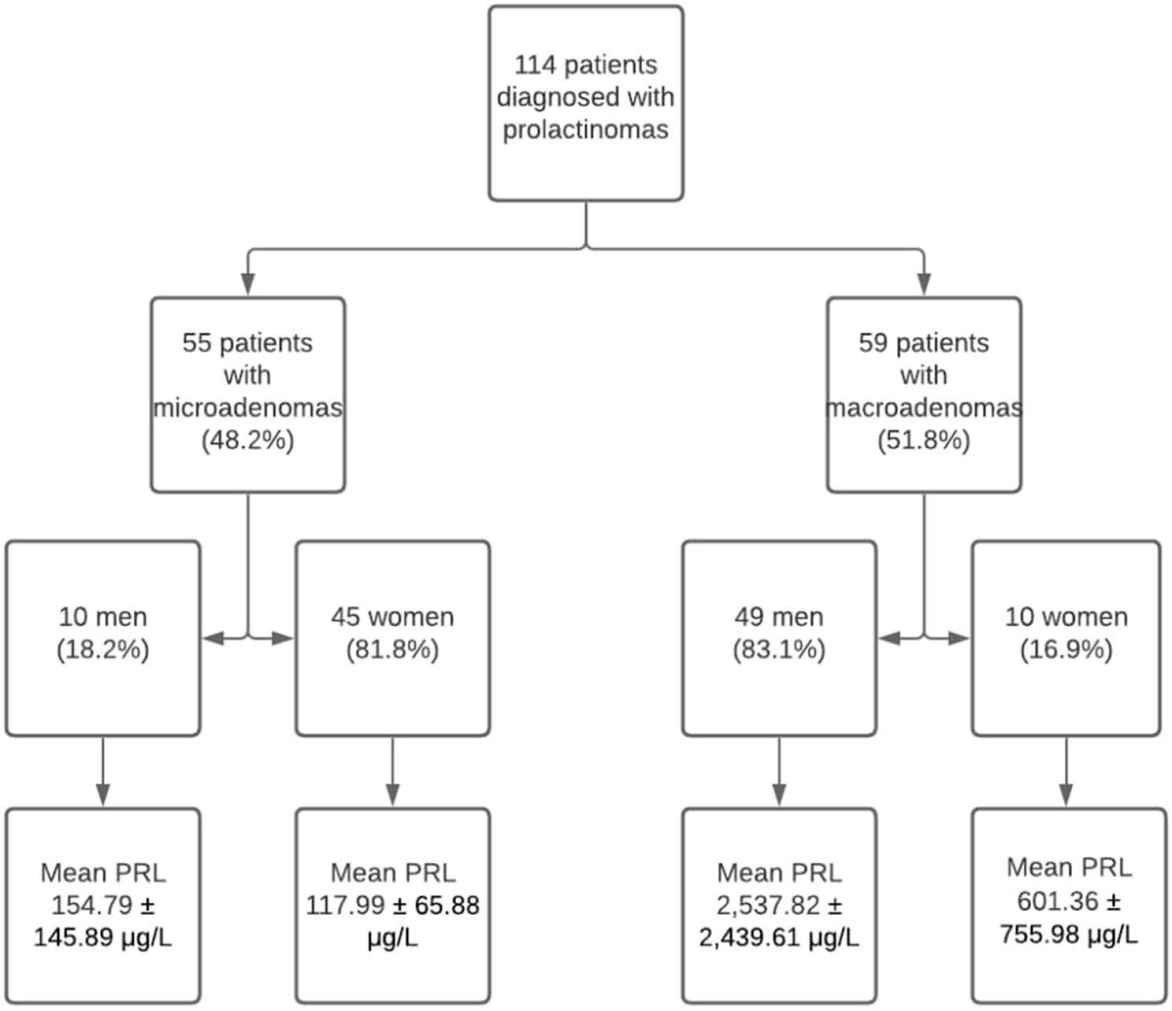
Study flow chart.

All patients included in the study underwent dedicated gadolinium enhanced magnetic resonance imaging of the hypothalamic-pituitary region (GE Healthcare) at diagnosis using thin slice, small field of view, dynamic contrast acquisition protocol, and according to the findings, tumours were defined as either microadenoma (longest diameter < 1 cm) or macroadenoma (longest diameter ≥ 1 cm). Size was defined as the maximum diameter in mm (Fig. 1).

### Ethical approval

The study received favourable ethical approval (GafREC ref: GF0403) by University Hospitals Coventry and Warwickshire (UHCW) Research & Development Governance Office, West Midlands, United Kingdom. All methods and experimental protocols were approved by and carried out in accordance with UHCW NHS Trust and National Institute for Health Research (NIHR) Good Clinical Practise (GCP) research guidelines and regulations. Informed consent was obtained from all subjects.

### Statistical analysis

**Figure 2:**
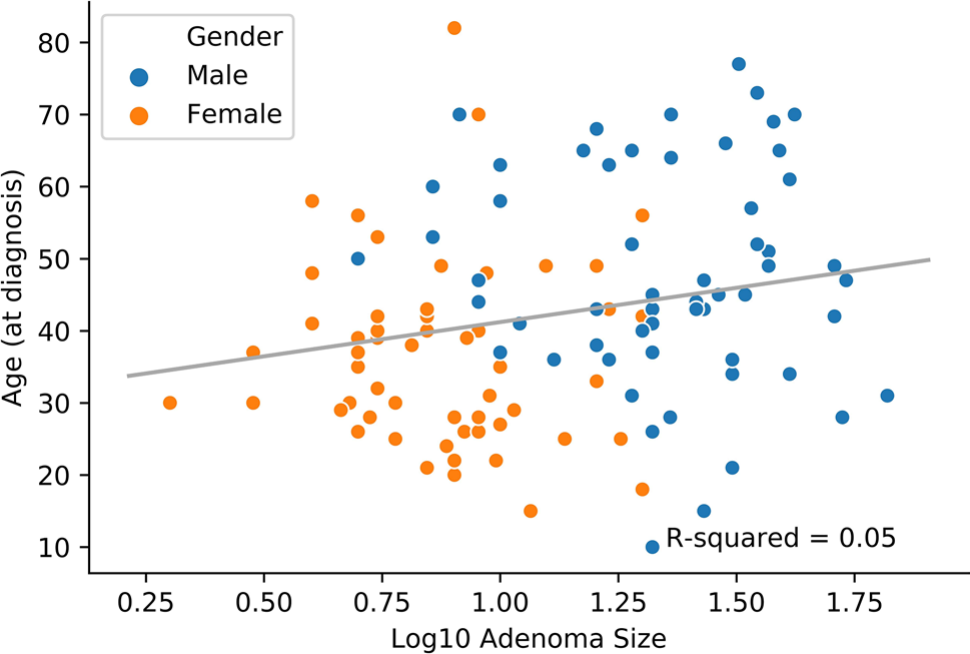
Correlation between age at diagnosis and adenoma size—grouped by gender.

**Figure 3:**
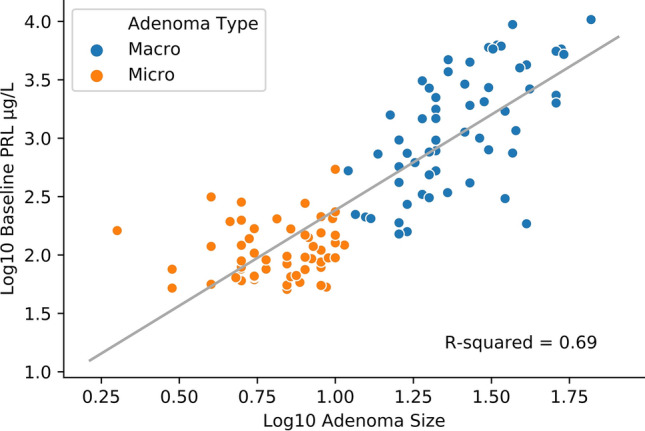
Correlation between baseline serum PRL concentrations and adenoma size—grouped by adenoma type.

Fully anonymised data of patients attending the dedicated Pituitary Clinic was collected using Microsoft Excel 2019, and analysed using Statistical Package for the Social Sciences Software (SPSS, IBM) version 20. The means and standard deviations (SDs) were calculated and compared using Independent Student t-test and for categorical variables we used Chi-squared test. Correlations were performed using Pearson’s analysis. A linear regression analysis was carried out in order to quantify the relationship between the size of the adenoma (dependent variable) and gender, age at diagnosis and baseline PRL concentration (independent variables). A probability value (*p* value) of < 0.05 was considered statistically significant. Receiver operating characteristic (ROC) curve analysis was performed to determine the cut-off serum prolactin concentration that could differentiate micro- from macroprolactinomas. Youden index was calculated by adding the sensitivity of the diagnostic test to the specificity, then subtracting 100 from the value. At a value of over 50%, the test can be administered for diagnostic purposes. Furthermore, the plots in Fig. 2, and 3, were created using Python version 3.7 (Python Software Foundation), and Spyder software (Integrated Development Environment), after normalising the data for baseline prolactin and adenoma size using the 10 base logarithmic function.

## Results

From the 114 patients included in the study, 51.8% were men, with a mean age at the time of diagnosis of 42.32 ± 15.04 (10–82) years. Of the female patients that were included in the analysis, 18.2% were post-menopausal. 48.2% of patients included in the analysis had microadenomas, and the mean tumour size (longest diameter in mm) was 16.54 ± 13.57 (3–65) mm. The baseline characteristics are summarised in Fig. 1, and Table 1.

**Table 1:** Patient characteristics according to tumour type.

Parameter	Microadenoma	Macroadenoma	*p* value
Age at diagnosis (years)	39.85 ± 13.88	44.63 ± 15.82	ns
Baseline prolactin (μg/L)	124.69 ± 85.37	2209.61 ± 2356.1	0.001
FSH (IU/mL)	5.64 ± 3.38	4.21 ± 7.38	ns
LH (IU/mL)	5.58 ± 3.58	3.99 ± 7.29	ns
Oestradiol (pmol/L)	161.45 ± 126.95	127.50 ± 57.07	ns
Testosterone (nmol/L)	4.87 ± 2.37	2.85 ± 2.64	ns
Adenoma size (mm)	6.05 ± 2.06	26.33 ± 12.37	0.001

*ns* not significant, *FSH* Follicle Stimulating Hormone, *LH* Luteinizing Hormone.

Significant differences were observed between male and female patients, and their baseline characteristics are summarised in Table 2. 9 patients (7.5%) presented with giant adenomas (largest dimension ≥ 40 mm), and all of them were male.

**Table 2:** Patient characteristics according to gender.

Parameter	Male	Female	*p* value
Age at diagnosis (years)	47.76 ± 14.97	36.49 ± 12.87	0.001
Baseline prolactin (μg/L)	2133.92 ± 2396.25	205.88 ± 366.31	0.001
FSH (IU/mL)	3.36 ± 3	6.92 ± 7.89	0.003
LH (IU/mL)	3.12 ± 2.72	6.90 ± 7.98	0.001
Oestradiol (pmol/L)	–	156.42 ± 119.01	–
Testosterone (nmol/L)	3.13 ± 2.68	–	–
Adenoma size (mm)	24.98 ± 13.81	7.49 ± 4.4	0.001
Macroadenoma (%)	81.8%	16.9%	0.001

*FSH* Follicle Stimulating Hormone, *LH* Luteinizing Hormone.

81.35% of men presented with hypogonadism, erectile dysfunction and decreased libido, while 79.1% of pre-menopausal women presented with oligomenorrhoea, and 18.51% had spontaneous galactorrhoea. None of the patients presented with neurological symptoms or visual field impairment caused by the mass effect of the macroadenoma.

**Figure 4:**
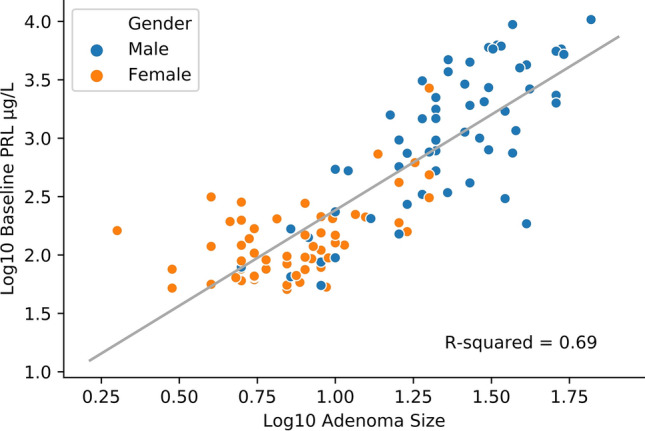
Correlation between baseline serum PRL concentrations and adenoma size—grouped by gender.

There were positive correlations established between the age at diagnosis and baseline PRL (r = 0.207, *p* = 0.027), and between age and adenoma size (r = 0.191, *p* = 0.042), the latest being presented in Fig. 2 In addition, baseline serum PRL concentrations were strongly correlated to the tumour dimension (r = 0.830, *p* = 0.001), as shown in Fig. 3 and 4.

After adding adenoma maximum diameter in mm and gender, age at diagnosis and baseline PRL concentration in a linear regression, correlations between tumour size and gender, and baseline PRL were maintained (*p* = 0.010 and 0.001 respectively), while age at diagnosis wasn’t associated with adenoma size (*p* = 0.519). Furthermore, a negative correlation between serum testosterone concentration in men and prolactinoma size was established (r =  − 0.438, *p* = 0.001), with lower testosterone in patients with larger tumours. In addition, hypogonadism was present in 81.35% of men, while remaining men experienced a significant decline in serum gonadotrophins.

**Figure 5:**
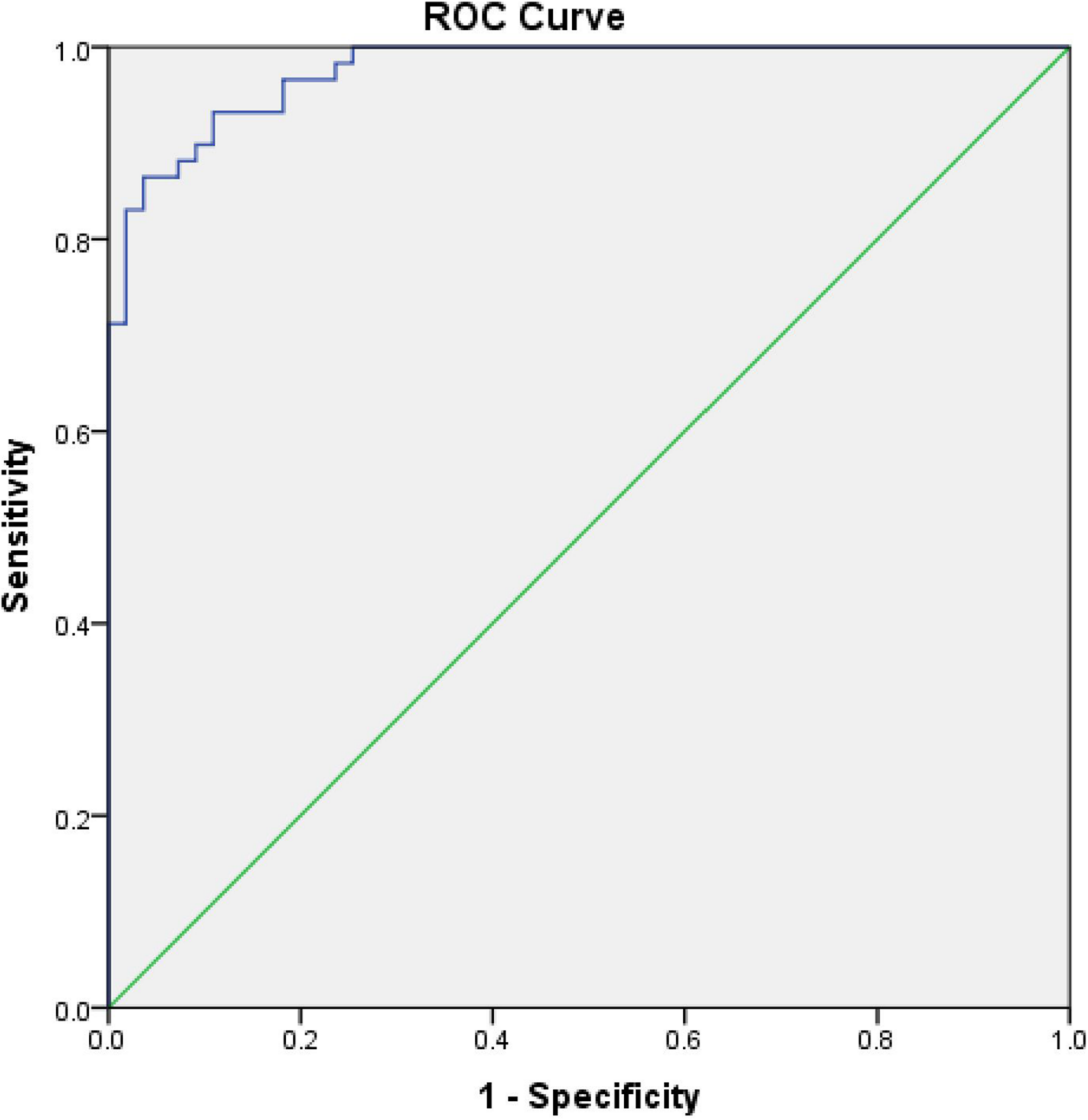
Receiver operating characteristic curve of *p* differentiation.

ROC curve analysis was performed to determine a concentration of PRL that could reliably differentiate between micro- and macro- prolactinomas (Fig. 5). The area under the curve was 0.976, indicating an excellent accuracy of the diagnostic method. For a cut-off PRL concentration of 204 μg/L (4338 mU/L), sensitivity and specificity were calculated at 0.932 and 0.891, respectively, with a Youden index of 82.3%.

## Discussion

In this retrospective cohort study, the biochemical characteristics of 114 patients diagnosed with prolactinomas were evaluated. Patients presenting with macroadenomas had no significant differences in age, had significantly higher serum PRL concentrations.

It is well established that prolactinomas display gender-based differences in accordance with the current study^2–5^. Women were younger than men at the time of diagnosis and were found to have microadenomas in 83.1% of cases while men had macroadenomas in 81.8% of cases. Mean adenoma size was significantly smaller in female patients, 7.49 ± 4.4 mm versus 24.98 ± 13.81 in males. Similar results were noted by Cander et al.^10^, with a mean adenoma size of 10.8 ± 9.4 mm in women, and 38.6 ± 21.6 mm in men. In that study, the prevalence of giant adenomas (> 4 cm maximum diameter) was higher, 14.2% (from which 81% were found in males) compared to 7.5% (100% in males) in our current study. Larger adenomas in men were also described by Delgrange et al.^11^ and by Nishioka et al.^12^, suggesting that gender is an important determinant of tumour size. The correlation between gender and baseline PRL was independent of the age at diagnosis, indicating that the high prevalence of large tumours in men is not caused by a delay in diagnosis, but by a higher frequency of rapidly growing tumours in this group of patients^10,11^.

Serum testosterone concentration in men was negatively correlated with adenoma size. Larger neoplasms were associated with a higher degree of gonadal dysfunction, while the prevalence of hypogonadism was 81.35%, comparable to the study of Tirosh et al.^13^, where pre-treatment prevalence of hypogonadism ranged between 75 and 90.9%, depending on the size of the prolactinoma.

The ROC curve analysis used to assess the cut-off of PRL threshold that could distinguish between a micro- and macro-adenoma showed a good performance, with area under the curve being 0.976. For a PRL cut-off value of 204 μg/L (4338 mU/L), both sensitivity and specificity of diagnosing a macro adenoma were optimal. While several studies assess the cut-off PRL value that could differentiate between a prolactinoma and a non-functioning pituitary adenoma^9,14,15^, and cut-off differentiating between a prolactinoma and “stalk effect” hyperprolactinemia, to the best of our knowledge, this is the first study reporting on a value that distinguishes micro- from macro- prolactinomas. In other studies, the cut off for distinguishing a true macroprolactinoma from a non-functioning pituitary adenoma (NFPA) was typically > 189 μg/L (> 4000 mU/L)^16^. Therefore, combining our data with previous studies we can suggest that a prolactin of > 235 μg/L (> 5000 mU/L) reliably distinguishes a macroprolactinoma from both micro adenoma and from an NFPA^1,9^.

Any patient with a true elevation in prolactin requires pituitary imaging. However, in the scenario of a younger patient with prolactin of 47–189 μg/L (1000–4000 mU/L) there is an ‘outside chance’ of an NFPA. In contrast, with a patient presenting with a PRL of > 204 μg/L (> 4338 mU/L) it is highly probable that the tumour will be a macroadenoma as this study highlights. This may be important in determining the urgency of radiological assessment and will help in managing the way this is communicated with the individual patient.

In patients with a high chance of macroprolactinomas referral for formal assessment of visual field defects and visual acuity can be organised in parallel with the imaging request. Furthermore, patients with macroadenomas are less likely to achieve sustained disease remission requiring closer monitoring after drug withdrawal^17,18^ and knowing this information early can facilitate physicians in managing patient expectations.

This study was conducted on a relatively large number of patients diagnosed with prolactinomas at a single academic institution. However, in order to identify a better cut-off value, the sample size needs to be increased. The study population was divided equally between both genders and both types of tumours, increasing the statistical significance of the results. It is noteworthy that 51.8% of the total cohort had macroadenomas. This is clearly at variance with the typical population prevalence of 20% of the prolactinomas being macroadenomas. This is likely due to a referral bias as our institution as a pituitary and neurosurgical centre would be referred a significant number of larger adenomas from a wider region whereas the majority of smaller tumours would be managed more locally. This does however permit more meaningful analysis as there is an even spread of tumour sizes and a large enough number of macroadenomas to achieve statistically significant differences.

### Limitations

The limitations of the current study consist in the retrospective analysis of data obtained from patients attending a single centre institution. In addition, a variance concerning the typical population of macroprolactinomas is observed in this study which may be perceived as a limitation by some and is likely due to a referral bias to a specialist pituitary service.

Finally, none of the patients included were resistant to dopamine agonist treatment, therefore the diagnosis of prolactinoma was not histologically confirmed.

## Conclusions

There has been much in the literature regarding a prolactin concentration which can distinguish NFPA from prolactinoma. There have also been multiple studies which have shown that the prolactin concentration is related to size of tumour and all patients with sustained hyperprolactinaemia go on to have pituitary imaging. This study however demonstrates for the first time that the degree of elevation of prolactin is a significant and useful parameter in predicting the size of the prolactinoma providing a reference prolactin concentration.

When a cut off PRL value of 204 μg/L (4338 mU/L) was used, specificity was calculated as 93.2%, and sensitivity as 89.1% in distinguishing a macro-adenoma from the more common micro-adenoma. It is therefore possible to make a confident diagnosis of a macroprolactinoma based on PRL levels of > 204 μg/L (4338 mU/L). This observation should be of clinical utility in discussing the likely pathology and management of the lesion with the patient in advance of the radiological studies.

## Data Availability

The data that support the findings of this manuscript are available by the corresponding author upon reasonable request.
